# Prevalence and correlations with depression, anxiety, and other features in outpatients with chronic obstructive pulmonary disease in China: a cross-sectional case control study

**DOI:** 10.1186/1471-2466-12-53

**Published:** 2012-09-10

**Authors:** Peian Lou, Yanan Zhu, Peipei Chen, Pan Zhang, Jiaxi Yu, Ning Zhang, Na Chen, Lei Zhang, Hongmin Wu, Jing Zhao

**Affiliations:** 1 Xuzhou Center for Disease Control and Prevention, 142 West Erhuan Road, Xuzhou, 221006, China; 2 Department of Respiratory Medicine, Affiliated Hospital of Xuzhou Medical College, 99 West Huaiai Road, Xuzhou, 221006, China

**Keywords:** Chronic obstructive pulmonary disease, Anxiety, Depression, Hospital Anxiety and Depression Scale

## Abstract

**Background:**

Patients with chronic obstructive pulmonary disease (COPD) often experience depression and anxiety, but little information is available regarding Chinese patients with these conditions. The present study assessed depression and anxiety in Chinese patients with COPD.

**Methods:**

A case–controlled study was designed with 1100 patients with COPD enrolled in the case group and1100 residents without COPD and respiratory symptoms selected as the control group. Anxiety and depression in both groups were evaluated using the Hospital Anxiety and Depression Scale (HADS). The body mass index,degree of airflow obstruction, dyspnea, and exercise capacity (BODE ) index was used to assess COPD severity. Binary logistic regression models were used to test the association between anxiety and depression.

**Results:**

The patients with COPD were more likely than controls to experience depression (cases, HADS 10.5 ± 3.6, prevalence 35.7%; controls, HADS 8.7 ± 2.7, prevalence 7.2%) and anxiety (cases, HADS 10.4 ± 3.1, prevalence 18.3%; controls, HADS 8.6 ± 2.1, prevalence 5.3%). Subjects with anxious and depressive symptoms had poorer health outcomes including a higher BODE index, a shorter 6-minute-walk distance (6MWD), more dyspnea, and a higher St George’s respiratory questionnaire (SGRQ) score. The prevalence of anxious and depressive symptoms increased with increasing BODE scores. On the basis of binary logistic regression, the BODE index was significantly correlated with anxiety (OR = 1.47, *p <* 0.001) and depression (OR = 1.51, *p <* 0.001). Anxious and depressive symptoms were also associated with several factors including younger age, female sex, higher education level, lower household income and history of smoking.

**Conclusions:**

This study confirmed the high prevalence of anxiety and depression in Chinese outpatients with COPD. Patients with COPD who had anxiety and/or depression had a poorer health-related quality of life.

**Trial registration:**

Chinese Clinical Trials Registration(ChiCTR-TRC-12001958)

## Background

Chronic obstructive pulmonary disease (COPD) is a leading cause of disability and death worldwide, with population prevalence rates of 5–13% [1-3]. Prevalence rates are directly related to tobacco smoking and indoor air pollution, and are expected to rise as smoking rates continue to increase, notably among women and in developing countries [1,3]. By 2030, COPD is expected to represent the third leading cause of death in middle-income countries [4]. COPD is characterized by poorly reversible limitations of airflow and dyspnea [5], but it is not limited to the lung. Some patients develop systematic manifestations, including exercise limitations [6,7], peripheral muscle dysfunction [6,8], and malnutrition [9]. Two of the most common and least-treated co-morbidities of COPD are anxiety and depression [10]. Increasing evidence suggests that anxiety and depression may have direct impacts on health status, hospitalization and exacerbation of COPD [11,12], rather than being consequences or markers of disease severity. Thus, detecting depression or anxiety in patients with COPD is of great importance. Although the close correlation between anxiety and depression is well known, few studies have examined their simultaneous occurrence in patients with COPD. Moreover, there are few studies assessing and comparing anxiety and depression levels among patients with COPD in China.

The aim of the present cross-sectional study was to investigate the levels and frequency of anxiety and depression among community-based patients with COPD. In addition, we evaluated the correlation between anxiety, depression, and other features on patient status with COPD in China.

## Methods

### Sampling strategy

This is a cross-sectional survey conducted between March and May 2008 in the rural areas of Xuzhou area. A cluster randomized sampling strategy was used. According to Chinese alphabetical sorting, one out of every nineteen communities was selected as a study community. Twelve communities were selected out of 238 communities. All patients in the primary care stations of the 12 selected communities were included. Based on age (±1 year), sex, education level, average number of family members and marital status, healthy participants who lived in the same villages as the patients with COPD were selected as control subjects. All sampled subjects were invited to participate in the study during home visits.

### Study population

Patients met the following criteria: (1) COPD diagnosed by the standards of the Global Initiative for Chronic Obstructive Lung Disease (GOLD); (2) no fever, no worsening of respiratory symptoms, no X-ray or medication changes within four weeks before recruitment; (3) no primary diagnosis of asthma; and (4) no previous lung volume reduction surgery, lung transplantation, or pneumonectomy. According to the sampling criteria, 1100 healthy participants were selected as control subjects.

### Demographic data

General characters such as age, sex, education level, average number of family members, marital status, smoking status (current-, ex- or never-smoker) and household net income were recorded based on patient reports. Subject weight and height were measured before carrying out pulmonary function tests and the body mass index (BMI) was calculated by dividing the weight in kilograms by height in meters squared. Education level was categorized as below high school, high school, or beyond high school education. Cigarette smoking was defined as having smoked at least 100 cigarettes in one**’**s lifetime.

### Assessment of anxiety and depression

Anxiety and depression were measured using the Hospital Anxiety and Depression Scale (HADS) [13,14]. The HADS has been specifically developed for detection of anxiety and depression in patients with somatic conditions. It is a validated screening tool for symptom severity in cases of anxiety and depression in patients with chronic diseases (including COPD) [11,12,14]. It is divided into an anxiety subscale (HADS-A) and a depression subscale (HADS-D) both containing seven items, rated 0–3, giving a possible maximum score for anxiety and depression of 21. Scores <8 indicate no clinical distress; scores from 8 to 10 indicate possible psychiatric morbidity; and scores ≥11 indicate probable pathologic levels of distress [13]. The Mandarin-Chinese HADS was also translated and validated in our population (unpublished data).

### Assessment of pulmonary function

Spirometry and bronchodilator response tests were carried out by trained professionals according to the standardized guidelines of the American Thoracic Society (ATS) [15]. Patients carried out the pulmonary function test at least 12 hours after the withdrawal of inhaled bronchodilators. Patients performed spirometry at 15 and 60 minutes after inhaling salbutamol (200 μg). GOLD stages were defined as described in the updated GOLD (2006 edition) [16].

### Assessment of quality of life

Health-related quality of 1ife (HRQL) was measured with Saint George’s Respiratory Questionnaire (SGRQ). The questionnaire is a widely used questionnaire specific to respiratory disease. It consists of 50 items and is separated into three parts: symptoms (distress due to respiratory symptoms), activities (effects due to impairment of mobility or physical activity), and effects (psychosocial effects of the disease) [17]. Scores were weighted using empirically derived weights. The scores range from 0 to l00 for the three subscales with a summary total score. Higher scores indicate worse health status; 0 indicates no impairment and 100 indicates maximal impairment [18].

### Assessment of dyspnea

Dyspnea was measured using the Medical Research Council (MRC) dyspnea scale. The MRC dyspnea scale classifies breathlessness into six grades (0 to 5) according to self-perceived breathlessness during daily activities [19].

### Assessment of exercise capacity

The six-minute walking distance test (6MWD) was carried out according to ATS guidelines. Each patient was ordered to walk as rapidly as possible in a solid and flat corridor for six minutes. The test was repeated twice with an interval of at least 30 minutes [20]. The better walking distances for each patient were recorded.

### Assessment of anxiety and depression impact on the health outcomes of patients with COPD

We used the validated BODE index, which is a multi-modal measure of disease severity, to assess COPD severity for each subject who was affected by anxiety and depression [21]. The BODE index is based on the BMI, the degree of airflow obstruction measured by forced expiratory volume in one second (FEV_1_), grade of dyspnea assessed by the modified MRC dyspnea scale [22], and exercise capacity measured by the 6MWD test. Each component is assigned a specific index and the total score ranges from 0 to 10 points (higher scores indicate greater severity). The BODE index predicts death and other poor outcomes with COPD [21,23,24]. The BODE index can be divided into four quartiles:quartile I is a score of 0–2; quartile II is a score of 3–4; quartile III is a score of 5–6; and quartile IV is a score of 7–10 [21,25].

### Statistical analysis

The computer-based analysis program Statistical Package for Social Science (SPSS) version 13.0 was used for all calculations. The minimal statistical significance level for all analyses was *p* <0.05. Group comparisons for categorical variables were performed using Pearson’s chi-square test or Fisher’s exact test. To compare groups of patients, we used ANOVAs for normally distributed data and the Kruskal–Wallis test for non-normally distributed data. For comparisons between two groups, Student’s *t*-test and the Mann–Whitney *U* test were used for normally and non-normally distributed data, respectively. Pearson’s correlation was used to assess the association between anxiety and depression. To evaluate the risk factors for depression, multivariate logistic regression analysis was performed, incorporating all factors that obtained values of *p* <0.05 in the bivariate analyses.

Ethical approval for the study was given by the Ethics Committee of the Xuzhou Center for Disease Control and Prevention, and the Regional Ethical Vetting Board, Xuzhou, China. Informed consent was obtained from all participants.

## Results

### General characteristics of participants

There were 1683 patients with COPD receiving care in the 12 selected communities. According to the patient screening criteria, 1145 patients (68.0%) met the screening criteria and 538 patients (32.0%) did not meet the screening criteria. Out of 1145 matching participants, 1100 matching participants had completed the questionnaires, while 45 paired subjects (12 patients, 33 controls) did not complete the questionnaires. There was no significant difference in age between responders and non-responders [mean age 62.0 (SD 11.6) and 61.8(SD 12.0) years, respectively; *p* =0.76].

The demographic characteristics of the 1100 healthy controls and the 1100 patients with COPD who completed the study are summarized in Table 1. The median age of all 2200 subjects was 62 years (range, 39 y to 76 y) and most (75.2%) were males. These values were similar for both the control and COPD groups. Disease severity according to the GOLD stage is shown in Table 2. The presence of dyspnea had the following distribution: 160 patients (14.5%), MRC 0; 465 (42.3%), MRC l; 355 (32.3%), MRC 2; 91(8.3%) MRC 3; and 29 (2.6%), MRC 4, with a median of 1 (P5-P95, 0–3). These patients showed a mean FEV_l_ of 1.27 L (SD, 0.36 L), a mean 6MWD of 445 m (SD, 91 m), a mean BMI of 23.3 k/m^2^ (SD, 2.8 k/m^2^), a mean SGRQ total score of 43.4 (SD, 17.6), and a median BODE index of 2 (P5-P95, l–6). Females manifested a significantly higher frequency of depressive symptoms (44.3% vs. 34.0%, *p* <0.001) and lower frequency of anxious symptoms (16.2% vs. 19.3%, *p* <0.001) than males (Table 3).

**Table 1:** Baseline characteristics of the study population according to disease status

**Variables**	**Groups**		***p*Value**
	**COPD (n = 1100)**	**Controls (n = 1100)**	
Number (Percentage, %)			
Female sex	273 (24.8)	273 (24.8)	≥0.05
Educated level to high school or beyond	21 (1.9)	21 (1.9)	≥0.05
Smoking history			
Past smoker	264 (24.0)	12 (1.1)	<0.05
Current smoker	226 (20.5)	235 (21.4)	
Never smoker	610 (55.5)	853 (77.5)	
HADS-A ≥ 8	201 (18.3)	58 (5.3)	<0.05
HADS-D ≥ 8	393 (35.7)	79 (7.2)	<0.05
Mean (SD) or median (quartile)			
Age	63.2 (40–75)	62.1 (39–76)	≥0.05
Average number of family members	2.86 (1–4)	2.86 (1–4)	≥0.05
Annual net income per family (yuan)	17002 (440)	17477 (469)	<0.05
BMI (kg/m^2^)	23.3 (2.8)	23.4 (3.0)	≥0.05
FEV_1_(L)	1.27 (0.36)	2.55 (0.68)	<0.05
FEV_1_, % of predicted value	45.2 (10.8)	88.6 (9.2)	<0.05
FEV_1_/FVC	47.9 (11.5)	83.6 (9.3)	<0.05
6MWD (m)	445.0 (91.0)	580.1 (99.8)	<0.05
HADS-A score	4.8 (2.3)	3.1 (1.6)	<0.05
HADS-D score	5.4 (2.6)	3.5 (2.4)	<0.05

HADS: Hospital Anxiety and Depression Scale: HADS-A: HADS—anxiety; HADS-D: HADS—depression; FEV_1_, forced expiratory volume in one second; FVC, forced vital capacity; BMI:body mass index; 6MWD: six-minute walking distance; MRC: Medical Research Council; SGRQ: Saint George’s Respiratory Questionnaire: BODE: body mass index, degree of airway obstruction, dyspnea and exercise capacity; Possible anxiety (8 ≤ HADS-A ≤ l0) and probable anxiety (HADS-A ≥ 11) were combined and referred to as anxiety (HADS-A ≥ 8). Possible depression (8 ≤ HADS-D ≤ l0) and probable depression (HADS-D ≥ 11) were combined and referred to and as depression (HADS-D ≥ 8).

**Table 2:** Depressive and anxious symptoms of the study population

**Variables**	**Healthy controls**	**COPD**				
		**I (n = 163)**	**II (n = 460)**	**III (n = 289)**	**IV (n = 188)**	**total**
HADS-A						
Total score	3.1 (0–12)	6.1(2–15)	6.3(4–17)	6.5(5–18)	7.0(6–19)^b^	6.7 (2–19)^a^
HADS-A ≥ 8	58 (5.3)	14(8.6)	70(15.2)	65(22.5)	52(27.7)^b^	201(18.3)^a^
HADS-D						
Total score	3.3 (0–13)	5.8(3–15)	6.5(5–17)	6.8(5–18)	7.3(7–20)^b^	6.7(3–20)^a^
HADS-D ≥ 8	79(7.2)	25(15.3)	118(25.7)	123(42.6)	127(67.6)^b^	393(35.7)^a^
BODE index	NA	2.4(1–6)	2.7(1–7)	3.3(1–8)	4.1(2–8)^b^	2.9(1–8)

HADS-A, HADS—anxiety; HADS-D,HADS—depression.

Values are expressed as the median (range) or number (%).

a *p <* 0.01 compared to healthy controls.

b *p <* 0.01 compared to mild-to-severe COPD.

**Table 3:** Disease-related characteristics of the COPD patients according to anxiety or depression status

	**Anxiety status**		***p*Value**	**Depression status**		***p*Value**
	**Yes (n = 201)**	**No (n = 899)**		**Yes (n = 393)**	**No (n = 707)**	
HADS-A score (mean, sd)	10.4(3.1)	3.5(2.1)	<0.05	NA	NA	NA
HADS-D score (mean, sd)	NA	NA	NA	10.5(3.6)	4.1(3.3)	<0.05
BMI (kg/m^2^) (mean, sd)	22.5(2.7)	23.7(2.8)	<0.05	23.4(2.9)	22.2(3.0)	<0.05
FEV_1 _(L)	1.29(0.35)	1.30(0.33)	>0.05	1.25(0.34)	1.29(0.35)	<0.05
6MWD (m) (mean, sd)	408.5(78.6)	456.8 (95.5)	<0.05	401.5(88.5)	460.6(100.5)	<0.05
MRC score (mean, 5th-95th)	2(0–4)	1(0–2)	<0.05	2(0–4)	1(0–2)	<0.05
BODE index (mean, 5th-95th)	4(1–8)	2.5(1–6)	<0.05	3.5(1–8)	2.5(1–6)	<0.05
SGRQ score (mean, 5th-95th)						
Symptom	72.5(19.5)	63.5(21.0)	<0.05	70.0(17.5)	62.0(18.7)	<0.05
Activity	66.3(16.8)	48.0(18.4)	<0.05	61.3(17.5)	47.8(19.5)	<0.05
Effect	56.6(18.2)	32.0(15.4)	<0.05	48.0(18.6)	30.7(18.8)	<0.05
Total	61.5(17.3)	40.6(16.4)	<0.05	55.3(16.2)	40.4(17.8)	<0.05
COPD severity (Percentage%)						
I	14(7.0)	149(16.6)	<0.05	25(6.4)	138(19.5)	<0.05
II	70(34.8)	390(43.4)	<0.05	118(30.0)	342(48.4)	<0.05
III	65(32.3)	224(24.9)	<0.05	123(31.3)	166(23.5)	<0.05
IV	52(25.9)	136(15.1)	<0.05	127(32.3)	61(8.6)	<0.05
Comorbid anxiety	NA	NA	NA	165(42.0)	36(5.1)	<0.05
Comorbid depression	145(72.1)	248(27.6)	<0.05	NA	NA	NA
Female sex	41(20.4)	232(25.8)	<0.05	112(28.5)	161(22.7)	<0.05

HADS: Hospital Anxiety and Depression Scale: HADS-A: HADS—anxiety; HADS-D: HADS—depression; FEV_1_, forced expiratory volume in one second; FVC, forced vital capacity; BMI:body mass index; 6MWD: six-minute walking distance; MRC: Medical Research Council; SGRQ: Saint George’s Respiratory Questionnaire: BODE: body mass index, degree of airway obstruction, dyspnea and exercise capacity; Possible anxiety (8 ≤ HADS-A ≤ l0) and probable anxiety (HADS-A ≥ 11) were combined and referred to as anxiety (HADS-A ≥ 8). Possible depression (8 ≤ HADS-D ≤ l0) and probable depression (HADS-D ≥ 11) were combined and referred to and as depression (HADS-D ≥ 8).

### Prevalence of anxious symptoms in patients with COPD

Table 1 shows the mean scores on the HADS-A for patients with COPD. The mean anxiety scores of patients with COPD were higher than those of healthy controls (*p* <0.05). Not only did the patients with COPD show a higher median anxiety score, but the patients with COPD were also significantly more likely than healthy controls to report anxious symptoms (patients: 18.3% vs. controls: 5.3%, *p* <0.01). Compared to the patients with COPD who had no anxious symptoms, the patients with COPD who had anxious symptoms had smaller means of BMI and 6MWD and had higher medians of MRC, BODE index, and SGRQ score (Table 3). In a subgroup analysis of patients with COPD, increased frequency of anxious symptoms was correlated with increased disease severity according to the GOLD stages (*χ*^2^ = 27.47, *p* <0.001) (Table 2). The prevalence of anxious symptoms also increased with increasing BODE quartiles (*χ*^2^ = 78.69, *p <*0.0001) (Table 2).

### Prevalence of depressive symptoms in patients with COPD

From Table 1, we knew that the HADS–D scores and frequency of depressive symptoms for the healthy controls and the patients with COPD differed significantly. The patients with COPD had higher HADS–D scores and greater frequency of depressive symptoms in comparison to the healthy controls (*p <*0.05). In comparison to the patients with COPD who had no depressive symptoms, the patients with COPD who had depressive symptoms had smaller means of BMI, FEV_1_, and 6MWD, and had higher medians of MRC, BODE index, and SGRQ score (Table 3). In a subgroup analysis of patients with COPD, increased frequency of depressive symptoms was correlated with increased disease severity according to the GOLD stages (*χ*^2^ = 133.03, *p* <0.001) (Table 2). The prevalence of depressive symptoms in patients with COPD increased as BODE index increased (*χ*^2^ = 102.34, *p <*0.0001) (Table 2).

### Relationship between anxiety and depression in COPD patients

According to the HADS scores, 165 (42.0%) depressive patients with COPD presented with concomitant anxious symptoms, and 145(72.1%) of the anxious patients presented with depressive symptoms (Table 3). Compared to patients without depressive symptoms, patients with depressive symptoms had significantly higher mean scores on the HADS (*p* <0.0001) (Table 3), and their symptoms of depression were positively correlated with anxious symptom (*r =* 0.701, *p <* 0.0001). The scores for anxious and depressive symptoms were correlated much more closely with the BODE index (r = 0.356, *p* <0.001; r = 0.386, *p <* 0.001; respectively) than with FEV_1_% (r = −0.072, *p* >0.05; r = −0.102, *p* >0.05; respectively).

### Determinants of anxiety and depression in patients with COPD

COPD was associated with a greater risk of anxious symptoms in both unadjusted (OR 4.02; 95% CI: 3.04 to 5.31) and adjusted multivariable analyses (OR 3.26; 95% CI: 1.69–5.36; see Table 4). Anxious symptoms were associated with several demographic and other personal factors including younger age, female sex, higher education level, lower household income, history of smoking, BODE index, SGRQ and comorbid depressive symptoms using a multivariate logistic analysis. However, no significant difference was observed between patients with or without anxiety with respect to FEV_1,_ FVC, or FEV_1_/ FVC (Table 4). This pattern of results was similar for control subjects without COPD. A multivariate logistic analysis identified six variables related to increased risk for clinically relevant depressive symptoms (*p* <0.05). These were younger age, female sex, higher education level, and lower household income, history of smoking, BODE index, SGRQ and comorbid anxious symptoms. However, no significant difference was observed between patients with or without depression regarding FEV_1,_ FVC, or FEV_1_/ FVC (Table 4). COPD was related to a higher risk of depressive symptoms in both unadjusted (OR 7.18; 95% CI: 5.67–9.10) and adjusted multivariable analyses (OR 3.58; 95% CI: 2.86–5.70; see Table 4).

**Table 4:** Risk of anxious and depressive symptoms in patients with COPD: multivariate logistic modeling

**Factors**	**Anxiety**			**Depression**		
	**Odds ratio**	**95% confidence interval (CI)**	***p***	**Odds ratio**	**95% confidence interval (CI)**	***p***
Age	1.16	1.12–1.19	0.000	1.13	1.05–1.20	0.003
Female gender	1.12	1.03–2.23	0.000	1.12	1.10–1.19	0.000
Education level	1.15	1.10–1.18	0.000	1.09	1.01–1.17	0.012
Household income	1.30	1.14–1.50	0.001	1.26	1.06–1.52	0.000
History of smoking	1.27	1.03–2.35	0.000	1.26	1.15–1.45	0.000
;FEV_1_	1.09	0.96–1.24	0.099	0.98	0.82–1.17	0.103
FVC	0.99	0.821–1.14	0.113	0.83	0.63–1.07	0.122
FEV_1_/FVC	0.97	0.88–1.08	0.102	1.02	0.94–1.09	0.098
BODE index	1.47	1.15–1.92	0.001	1.51	1.13–1.95	0.000
;SGRQ	1.35	1.11–1.87	0.000	1.47	1.12–2.01	0.000
Depression	3.26	1.69–5.36	0.000			
Anxiety				3.58	2.86–5.70	0.000

FEV_1_, forced expiratory volume in one second; FVC, forced vital capacity; BODE: body mass index, degree of airway obstruction, dyspnea and exercise capacity; SGRQ: Saint George’s Respiratory Questionnaire.

### Anxiety and depression and COPD-related outcomes

Anxiety and depressive symptoms were associated with poorer patient-centered outcomes. Table 3 shows that anxious patients had a higher proportion of concurrent depressive symptoms, a shorter 6MWD, more severe dyspnea, worse SGRQ, a higher COPD severity and a higher BODE index as compared with non-anxious patients.Similar results were found for depressed patients compared with non- depressed patients with respect to concurrent depression, 6MWD, dyspnea, SGRQ and the BODE index. Higher COPD severity, as measured by the BODE index, was associated with a greater risk of anxiety and depressive symptoms among patients with COPD (Table 2). The BODE index and concurrent anxious symptoms were related to depressive symptoms, whereas the BODE index and concurrent depressive symptoms were associated with anxious symptoms. For each unit increase in the BODE index, the anxiety risk increased by 56.2% (OR 1.72; 95% CI: 1.28–2.31), and the depression risk increased by 48.7% (OR 1.56; 95% CI: 1.23–2.01). Co-morbid anxious symptoms increased the risk for depressive symptoms by 36.9% (OR 3.25; 95% CI: 1.67–5.35), and co-morbid depressive symptoms increased the risk for anxious symptoms by 44.5% (OR 3.56; 95% CI: 2.85–5.68).

The multivariate logistic regression model was used to explore which components of the BODE index were independently associated with anxious and depressive symptoms. The FEV_l_%, BMI, MRC and 6MWD were used as the independent variables. The results showed that MRC and 6MWD were independent predictors for anxious symptoms and FEV_l_% and MRC were independent predictors for depressive symptoms (Table 5).

**Table 5:** Multivariate logistic regression of the components of the bode index on anxious and depressive symptoms

**Variables**	**Anxiety**			**Depression**		
	**Odds ratio**	**95% confidence interval (CI)**	***P***	**Odds ratio**	**95% confidence interval (CI)**	***P***
BMI (kg/m^2^)	0.953	0.871~1.043	0.105	0.973	0.896~1.057	0.123
FEV_l_%	0.989	0.946~1.028	0.099	1.25	1.16~1.34	0.000
6MWD (m)	1.51	1.39~1.64	0.000	0.995	0.991~1.08	0.159
MRC	2.04	1.76~2.36	0.000	2.01	1.77~2.29	0.000

BMI, body mass index;FEV_1_, forced expiratory volume in one second;6MWD: six-minute walking distance; MRC: Medical Research Council.

## Discussion

The present study found a higher prevalence of depressive and anxious symptoms among patients with COPD in China compared to a matched healthy control group without the condition, while the scores for anxious and depressive symptoms were higher among the patients with COPD than among the controls. Previous studies have reported anxiety symptoms ranging from 9.57–49%, and depressive symptoms ranging from 22.8–52%, using the HADS as the screening tool [12,26,27]. Our findings confirmed a higher frequency of anxious (18.3%) and depressive symptoms (35.7%) in stable COPD patients. Higher percentages of patients with COPD than of controls reported symptoms of depression, while anxiety was also more common in the patients than in the controls. These high rates stress the importance of screening and treatment of depression and anxiety in patients with COPD to maintain health-related quality of life [28].

Of importance was our result that more COPD patients with anxiety had depression than COPD patients with depression had anxiety. Given that there was a significant correlation between anxiety and depression, our result should remind general practitioners that when patients have symptoms of anxiety or depression, the other condition should be suspected as well. The prevalence of anxious and depressive symptoms increased with the GOLD stage and BODE index, as established in previous studies [29-31]. These corresponding increases suggest that when the general practitioner detects poorer health status in patients with COPD, they should consider giving psychological guidance along with medical treatment.

The present study showed a correlation between depressive or anxious symptoms and age, sex, education level, household income, history of smoking, BODE index, SGRQ and co-morbid anxious or depressive symptoms, which was partially consistent with the results of previous studies [27,31-33]. This is of relevance for practice: general practitioners need to be aware that anxiety and depression are common conditions in COPD patients. The present result found that patients who were younger, females, a higher education level, lower household income, higher BODE index, higher SGRQ scores and a history of smoking suffered from anxious or depressive symptoms more frequently. The relationship of age to anxiety and depression in patients with COPD has been investigated by Cleland *et al.*[28]. A previous study has reported susceptibility to depression in female patients with COPD [32]. Consistent with this result, our data confirmed that the frequency of depression in female patients with COPD differed significantly from that in male patients with COPD, unlike the report from Cleland *et al.*[28]. This is likely due to different study populations. The majority of women in rural areas in China are housewives, and when they become ill, it means that the family has no food to eat, which may produce more anxiety and depression. The increase in anxiety and depression with advancing educational level could be due to the lower education level of our participants; they were all junior high school level. Their understanding of COPD is less, so they may be more prone to anxiety and depression. The COPD patients who had low family income tended to suffer from anxiety and depression, perhaps because there were no local medical treatment safeguard measures. Patients with a higher degree of airflow limitation reported more symptoms of depression and increased risk for depression, and pulmonary functioning was found to be a predictor of depression [34,35]. However, FEV_l_ alone was not capable of predicting the presence of anxiety or depression [12]. In the present study, the FEV_l_ had no clear relation with anxiety or depression scores. The BODE index had a stronger association with anxious and depressive symptoms than did the FEV_l_ in our study, consistent with the results of previous studies [21,26,27,35]. Our results also confirmed that the SGRQ was related to anxiety and depression in the patients with COPD. This revealed that the decline in the quality of life can lead to the generation of anxiety and depression, and vice versa, anxiety and depression can lead to reduced quality of life.

We found a strong positive correlation between BODE index and GOLD stage. Although considerable overlap between stages was also found within BODE quartiles, the BODE index can indeed be used to discern groups of COPD patients. This suggest that the BODE index could better reflect the anxiety and depression status of patients with COPD [36].

Our data indicated that subjects who were anxious and depressive walked a shorter six-minute walking distance (6MWD), had more dyspnea, a higher BODE index, and lower SGRQ. The anxiety and depression scores and the prevalence of anxious and depressive symptoms increased as BODE quartiles increased. From the binary logistic regression results, the BODE index was an independent predictor for anxious and depressive symptoms after being adjusted for other confounders. To explore the association between anxious and depressive symptoms and the components of the BODE index, a binary logistic regression model was used to analyze the relation. The results showed that anxiety was associated with shorter distances walked (6MWD)and dyspnea (MRC); however, depression was associated with FEV_1_% and more dyspnea (MRC).

Although this is, to date, the largest study to evaluate the frequency and risk factors associated with depression and anxiety in Chinese patients with COPD, it has certain limitations. Although we used a reliable and valid measure of anxiety and depression, our measure was not a clinical diagnosis of a generalized anxiety and depression disorder. We were also not able to evaluate changes in anxiety and depression over time. In addition, there is the possibility of misclassification of COPD, although we took rigorous steps to avoid this.

## Conclusion

Patients with COPD are at increased risk for depressive and anxious symptoms. The patients who had younger age, female sex, higher education level, lower household income, higher BODE index, higher SGRQ scores and a history of smoking were more likely to suffer from anxious or depressive symptoms. Subjects who were anxious and depressive walked a shorter six-minute walking distance (6MWD), had more dyspnea, a higher BODE index, and lower SGRQ. The present study adds evidence for the usefulness of the BODE index to assess how anxious and depressive symptoms impact the health outcomes of patients with COPD. The BODE index was shown to be closely related to anxious and depressive symptoms in stable COPD patients. Contrary to previous classification systems based only on FEV_l_, COPD severity assessed by the BODE index can be more directly related to self-perceived symptoms (dyspnea), difficulties with activities of daily life, and physical mobility (exercise capacity), which were the risk factors for anxious and depressive symptoms. The BODE index should be included when assessing COPD severity given that anxiety and depression are significant co-morbidities of COPD and can be treated.

Our findings show the importance of screening for depressive and anxious symptoms in patients with COPD, particularly those with younger age, higher SGRQ, higher BODE index, or history of smoking, so as to provide adequate patient care. Further studies are needed to elucidate the effectiveness of treating psychiatric distress in these patients, as well as the efficacy of screening to identify those who might benefit from specific therapy.

## Competing interests

The authors declare that they have no competing interests.

## Authors’ contributions

PL participated in title and abstract screening, full text screening, and contributed to the manuscript drafts. YN conceived of the study, participated in the design of the study, title and abstract screening, full text screening, data extraction and analysis, and drafted the manuscript. PC carried out the grey literature search, participated in title and abstract screening, full text screening, and contributed to the manuscript drafts. PZ and JY conceived of the study, participated in the design of the study, title and abstract screening, full text screening, and contributed to the manuscript drafts. NZ and NC contributed to the conception of the study, participated in the study design, and contributed to the manuscript drafts. LZ, HW and JZ were the lead authors on the original review, contributed to the conception of the study, participated in the study design, and contributed to the manuscript drafts. All authors read and approved the final manuscript.

## Pre-publication history

The pre-publication history for this paper can be accessed here:

http://www.biomedcentral.com/1471-2466/12/53/prepub
